# Social Preference of Children at Risk for ADHD in Schools: Do They Have Limited Social Resources and can Friends Protect Against Peer Rejection?

**DOI:** 10.1177/10870547251349244

**Published:** 2025-06-27

**Authors:** de Swart F., Veenman B., Oosterlaan J., Luman M.

**Affiliations:** 1 Vrije Universiteit Amsterdam, Clinical, Neuro- and Developmental Psychology, Amsterdam, The Netherlands; 2 Accare Child Study Center, Groningen, The Netherlands; 3 Emma Children’s Hospital, Amsterdam UMC, University of Amsterdam, Emma Neuroscience Group, Department of Pediatrics, Amsterdam Reproduction & Development, Amsterdam, The Netherlands

**Keywords:** ADHD behaviors, peer relationships, friendships, social preference, primary education

## Abstract

**Objective:**

This cross-sectional study investigated social opportunities in children at risk for ADHD in regular primary education classrooms. First, we aimed to compare social preference of children at risk for ADHD and of their unilateral friends, with their classmates. Second, we hypothesized that for the at risk-group higher levels of problem behaviors would be related to having friends with lower social preference, via own lower preference (limited social resources-hypothesis). Third, we hypothesized that having friends with higher social preference could buffer against lower social preference of children in the at-risk group (interpersonal contact-hypothesis).

**Method:**

Our sample consisted of 112 Dutch primary school children at risk for ADHD (84% boys, *M*_age_ = 8.89, *SD* = 1.85), and 2,526 classmates serving as controls. Social preference and preference of unilateral friends was assessed with peer nominations of friendships and peer ratings of preference. Behavioral problems of children at risk for ADHD were assessed by teacher questionnaires.

**Results:**

T-tests revealed that children at risk for ADHD and their unilateral friends had lower social preference than their classmates. Children at risk for ADHD nominated classmates with higher preference than themselves as friends, while they were nominated by classmates with similar preference. A path model and two linear regression models showed that higher levels of conduct problems were indirectly related to lower social preference of received unilateral friends, via own lower preference. We found no evidence that interpersonal contact could protect against lower social preference.

**Conclusion:**

Clearly, children at risk for ADHD suffer from problems in their relationships with classmates.

In primary school, every classroom has one or two children with attention-deficit/hyperactivity disorder (ADHD) (5%, Sayal et al., 2018), and an even larger percentage (up to 15%) of children are at risk for ADHD showing elevated—but subclinical—levels of ADHD symptoms (Wåhlstedt et al., 2008). At school, children with inattentive, hyperactive and/or impulsive behavior (symptoms of ADHD) often display problems in the interaction with classmates (e.g., sharing, cooperation, and turn-taking) or show behaviors that are not in line with classroom-wide rules (e.g., blurting out answers before a question has been asked; Diamantopoulou et al., 2007; Gardner & Gerdes, 2015). These behavioral problems pose an increased risk for the classroom relationships of children at risk for ADHD, leading to peer rejection by classmates and having lower quality friendships (Diamantopoulou et al., 2005; Farmer et al., 2018; Grygiel et al., 2018; Hoza et al., 2005). The high prevalence of children at risk for ADHD in classrooms suggests that there may be many children in need of classroom support in relation to social functioning.

Moreover, the social opportunities of children with ADHD behaviors are not only affected by their own characteristics, but also by their classroom social context. On the one hand, well-liked classmates might not be available as friendship options for children at risk for ADHD, and therefore they might resort to friendships with classmates who are disliked as well (Ettekal & Ladd, 2015). On the other hand, affiliating with well-liked classmates might protect against being rejected by peers (J. K. Dijkstra et al., 2010). Since being liked by peers and having high quality friendships are beneficial for children’s adaptive behavior and wellbeing (e.g., Bukowski et al., 2018; Gifford-Smith & Brownell, 2003), insight in these social processes is important. Indeed, in general wellbeing is found to be lower for children with ADHD (Peasgood et al., 2016). The aim of this study is to get more insight into the classroom relationships with peers of children at risk for ADHD.

## Social Preference and Friendships in Children at Risk for ADHD

Children with ADHD behaviors often experience difficulties in their relationships with classmates, such as low social preference and lower quality friendships (Hoza et al., 2005). Social preference is a measure of social status that describes the degree to which children are liked, or accepted by their classmates, opposed to popularity which is a status measure based on social visibility and reputation (Bukowski et al., 2018; Gifford-Smith & Brownell, 2003). Friendships, in turn, are dyadic social relationships between children. Next to dyadic friendships two types of unilateral friendships can be distinguished: given friendships and received friendships (Lodder et al., 2017). Given friendships pertain to friends that the child selects, but that are not reciprocated by the selected classmate. Received friendships pertain to friends that the child is selected by, but which are not reciprocated by the child. Both dyadic and unilateral friends fulfill social provisions of children (Yust et al., 2023). For the current study our focus was on both types of unilateral friends, as they provide a more nuanced view of children’s classroom affiliations.

For a substantial proportion of children with ADHD behaviors, difficulties in classroom relationships may be explained by additional behavioral problems including oppositional, defiant, and/or aggressive behaviors and/or problems with anger-regulation (Mikami, 2010). In addition, these children often experience social skill problems, such as difficulties in cooperating, sharing or turn-taking (Gardner & Gerdes, 2015; Mikami, 2010; Mikami & Normand, 2015) and they show more difficulties providing emotional support and resolve interpersonal conflicts in friendships (McKee, 2017). These problems make this group of children less desirable as playmates and friends (McKee, 2017) and may lead to lower social acceptance by peers and lower quality friendships than typically developing classmates (e.g., Cardoos & Hinshaw, 2011; Gardner & Gerdes, 2015). A study by Hoza et al. (2005) indeed shows that 56% of children with ADHD had no dyadic friendship, and 52% were rejected by classmates, compared to respectively 32% and 14% in comparison children. Moreover, when ADHD behaviors are more severe, peer rejection is higher and friendship quality is lower (de Swart et al., 2019; McKee, 2017; Normand et al., 2023).

## Limited Social Resources?

Based on the similarity attraction theory, people tend to seek out friends who are similar to them in behavioral characteristics, gender, or cultural background (Gifford-Smith & Brownell, 2003). It is commonly acknowledged that children with high levels of aggression seek out similarly aggressive children as friends (Henry et al., 2000; Sijtsema et al., 2010). Research suggests that this may also be true for children at risk for ADHD (McKee, 2017) as they seem to be friends with other children who show similar behaviors (Mariano & Harton, 2005). However, friendship selection may also take place based on similarity in social status (e.g., Ettekal & Ladd, 2015; Farmer et al., 2018; Laursen, 2017). In fact, research shows that differences in peer acceptance are a predictor for the dissolution of friendships (Hartl et al., 2015). Thus, children that are rejected by peers may befriend other peers who are rejected as well (see Hooijsma et al., 2020; Scholte et al., 2009). This may in part be an active effort based on similarity, but also an involuntary result of the fact that classmates that are more liked might be less available as friends (see Farmer et al., 2018). Classmates might not want to befriend children at risk for ADHD due to negative, or even stigmatizing views of these children (Mikami, Na, et al., 2022; Mikami & Normand, 2015), limiting their access to social resources.

Ample research suggests that an important explanation for the limited social resources of children that are disliked is that classmates that befriend a rejected peer may risk rejection themselves (see e.g., Farmer et al., 2018; Hartl et al., 2015; Sentse et al., 2013). These reputational costs can lead classmates to avoid associating with a rejected peer. Consequently, higher levels of problems of children at risk for ADHD and subsequent peer rejection, may result in the selection of friends (given friendships) with lower social preference, and being selected by children with lower social preference (received friendships; see Scholte et al., 2009). There is some evidence for this in a study that showed that children with ADHD were less likely to be nominated as a friend by highly preferred classmates (Hoza et al., 2005). Thus, further insight is needed into how levels of problems of children at risk for ADHD are indirectly related to the social preference of their friends, via their own social preference.

## Protective Effects of Interpersonal Contact?

While children at risk for ADHD may have limited social resources, the social resources that are there, such as having a well-liked friend, could buffer against peer rejection that may be the result of their levels of problems. Intergroup contact theory (see Pettigrew & Tropp, 2006) suggests that whom one’s friends are may play an important role in the extent to which children are rejected by their classmates (J. K. Dijkstra et al., 2010; Y. H. M. Van den Berg & Stolz, 2018). A phenomenon described as “basking in reflected glory” describes that particularly affiliation with friends with higher social status has positive consequence for one’s own status (Cialdini & Richardson, 1980, p. 406; J. K. Dijkstra et al., 2010). Desiring to be popular or gaining affection (liking) from others is an important reason for children to affiliate with classmates with higher status (J. K. Dijkstra et al., 2010; Gifford-Smith & Brownell, 2003; Rubin et al., 2006). Research among adolescents found that being liked by (but not being the best friend of) a popular classmate increased likeability (J. K. Dijkstra et al., 2010). Moreover, rejected children who were seated next to a well-liked classmate in a classroom experiment were judged more favorable by that classmate after the experiment (Y. H. M. van den Berg & Stolz, 2018). Thus, for children at risk for ADHD being affiliated with a well-liked classmate might increase their social preference in the classroom, compared to those having friends who are less well-liked. Consequently, having a well-liked friend could moderate the relationship between children’s levels of problem behaviors and peer preference.

## Present Study

The aim of this cross-sectional study was to test social processes that may limit or promote social opportunities of children at risk for ADHD in primary school classrooms. First, we investigated whether children at risk for ADHD, compared to typically developing classmates, displayed lower social preference (Gardner & Gerdes, 2015; Hoza et al., 2005), and have lower preferred given as well as received friends (hypothesis 1; Scholte et al., 2009). Exploratorily, we compared social preference of children in the at-risk group to their given and received friends’ preference. Next, we tested two hypotheses on social classroom processes for children in the at-risk group, based on limited social resources theory and intergroup contact theory. We hypothesized that higher levels of behavioral problems (i.e., inattentive behavior, impulsive/hyperactive behavior, conduct problems) would indirectly be related to having lower preferred friends, via children’s own lower social preference (i.e., limited social resources-hypothesis; hypothesis 2). Furthermore, we hypothesized that friends’ preference would moderate associations between higher levels of behavioral problems and lower peer preference (i.e., intergroup contact-hypothesis; hypothesis 3).

## Method

### Participants

The study sample comprised 112 children (84% boys) in the age of 6 to 13 years (*M* = 8.89, *SD* = 1.85) that showed significant and impairing levels of ADHD behaviors in the classroom (from now on, the at risk-group; although children with a diagnosis of ADHD were not excluded), and 2,526 classmates who served as comparison group and as raters of the peer nomination data (average participation rate per classroom = 97%). Participants came from 85 classrooms of 60 regular primary schools. Children at risk for ADHD were included if they displayed (a) high levels of ADHD symptoms (>90th percentile) on the teacher-rated Inattention and/or Hyperactivity/Impulsivity scale of the Disruptive Behavior Disorders Rating Scale (DBDRS, Dutch version; Oosterlaan et al., 2008) and (b) at least four ADHD symptoms as assessed by Teacher Telephone Interview (TTI; Holmes et al., 2004), a semi-structured interview based on Diagnostic and Statistical Manual of Mental Disorders (DSM-5; American Psychiatric Association, 2013). The TTI cut-off criterion of four ADHD symptoms was used because the presence of at least four ADHD symptoms is related to significant psychosocial impairments (Kooij et al., 2005). Children were excluded if they: (a) suffered from a neurological or severe physical condition interfering with daily functioning; (b) had an IQ score lower than 80, estimated using a two-subtest short version of the Wechsler Intelligence Scale for Children-third edition (WISC-III,^1^ including Block Design and Vocabulary; Sattler, 1992), or (c) received any ADHD treatment or behavioral teacher program at study entry or in the preceding month, because the current study was part of a larger study assessing the effectiveness of a behavioral teacher training program (Veenman et al., 2016). No more than two children in the at-risk group per classroom and five classrooms per school were allowed to participate to limit teacher burden and to increase heterogeneity of teacher and school settings involved (Scherbaum & Ferreter, 2009). Sample characteristics are described in Supplement 1. The large majority of our sample (91%) involved children without a clinical diagnosis of ADHD.

A total of 271 people (teachers, parents and professionals such as school counselors) requested information on study participation. For 152 children, written informed consent was obtained, after which eligibility of these children was assessed. Two children were excluded based on the teacher-rated DBDRS, two based on the TTI, three for receiving a (pharmacological) treatment for ADHD, and two for having a neurological condition. Fifteen children were excluded because the maximum of two per classroom and five classes per school was reached, and 14 children due to the use of a classroom program interfering with the behavioral program of the effectiveness study. Finally, three students were excluded because their teachers were opposed to the administration of sociometric measures in their classroom.

For 2,526 classmates (comparison group) peer nomination data was gathered. No other information was available for these classmates. There were no missing data for peer nominations, and for peer ratings this was 1.8%. For the at-risk group, the percentage of missing data for the SWAN and the SDQ was 3.6% and 1.8%, respectively.

### Measures

**Behavioral problems** Inattentive and hyperactive/impulsive behavior were assessed with respective subscales of the teacher version of the Strengths and Weaknesses of ADHD-symptoms and Normal Behavior (SWAN; Swanson et al., 2006). Both subscales consist of 9 items, which are scored on a 7-point Likert scale (−3 = far above average, +3 = far below average; Young et al., 2009). Examples of items are “sustain attention on tasks and play activities” and “organize tasks and activities” for inattentive behavior, and “settle down and rest (control constant activity)” and “modulate verbal activity (control excess talking)” for hyperactive/impulsive behavior. Items were reverse scored so that higher scores indicated more problems. Internal consistency for the teacher SWAN in the current study was high (α = 0.91). Convergent validity of the SWAN is adequate (*r* = 0.54 with the Hyperactivity/Impulsivity scale of the Strength and Difficulties Questionnaire; Lakes et al., 2012).

Conduct problems was measured with the respective scale of the teacher version of the Strengths and Difficulties Questionnaire (SDQ, Dutch version; Goedhart et al., 2003). The subscale comprised of five items, and was rated on a 3-point Likert scale (0 = not true, 1 = somewhat true, and 2 = certainly true). Examples are “Often has temper tantrums or hot tempers” and “Often lies or cheats.” The internal consistency of the teacher SDQ scales (Dutch version) is satisfactory (0.74 < α < 0.81) and the scales have adequate concurrent validity with other measures of psychopathology (Van Widenfelt et al., 2003).

**Social preference** Social preference was assessed with peer ratings. Peer ratings are a reliable way to assess social preference (Bukowski et al., 2018). To obtain peer ratings, all classmates of participating children were presented a list with names of all classmates. Children rated their classmates on a 5-point Likert scale (1 = dislike very much, 3 = neutral, 5 = like very much; Hoza et al., 2005). Explanatory faces (from frowning to smiling) were placed above the numbers to facilitate the interpretation of the numbers. To calculate social preference scores, we transformed the one-dimensional rating scale into two dimensions of respectively acceptance and rejection following procedures of Maassen et al. (1996). This is an accepted method, since children will rarely nominate other children at the same time as liked and as disliked (Maassen et al., 1996). First, we transformed the Likert scale into −2 (=dislike very much) to 2 (=like very much). Rejection ratings were then transformed to absolute values (0, 1, 2), higher scores indicating more rejection by peers. For each participant, the sum of the ratings received for acceptance and rejection were now calculated separately. Acceptance and rejection scores were standardized (z-scores) within classroom. Social preference scores for children in the at-risk group were obtained by subtracting rejection ratings from acceptance ratings, after which scores were again standardized.

**Friends’ social preference** We assessed social preference of unilateral friendships (i.e., given and received friendships). To assess social preference of given friends, children were asked to write down the names of three classmates whom they considered their first, second, and third best friends. The average of the social preference scores of the nominated friends was utilized as an indication of given friends’ social status. To assess social status of received friends, the average social preference scores of the peers that nominated the child as first, second, or third best friend were calculated (Hoza et al., 2005).

### Procedure

This study was carried out in the Netherlands between September 2011 and July 2014 as part of a larger study investigating the effectiveness of a behavioral teacher program for children that show significant and impairing levels of ADHD behaviors in the classroom (Veenman et al., 2016). Dependent measures for this study were obtained at baseline, prior to the start of the intervention study. Procedures were approved by the local medical ethical committee (reference number 2011/196, Vrije Universiteit Medical Centre). Teachers and parents were recruited through educational consultant associations, the national parent association for children with developmental problems, and the study’s website. Teachers who were interested in participating, enlisted one or two children displaying elevated ADHD behaviors in their classroom for whom they wanted to use the behavioral teacher training program. For all participants, written consent was obtained from teachers, parents, and children older than 11 years. After a participant was included in the study, parents of participant’s classmates received an information letter explaining the study and an offer to opt out (3%). For children in grade 1 and 2, sociometric measures were administered individually in a quiet room, to assure they understood the instructions and were not overheard by classmates. All other children received instructions in their classroom with tables separated from each other to allow private ratings. Teachers completed the SWAN and SDQ online. Demographic information was gathered from parents.

### Data Analyses

To determine whether social preference and friends’ preference of children at risk for ADHD differed from their classmates (hypothesis 1), we calculated whether z-scores of children at risk for ADHD differed significantly from zero (i.e., the score of the average classmate), utilizing a one-sample t-test in SPSS. Cohen’s *d* was utilized to calculate effect sizes, with values of 0.20, 0.50, and 0.80 as thresholds of small, medium, and large effect sizes, respectively (Cohen, 2013). Preference differences between own preference, preference of given friends, and of received friends within the at-risk group were investigated with paired sampled t-tests.

**Figure 1.**
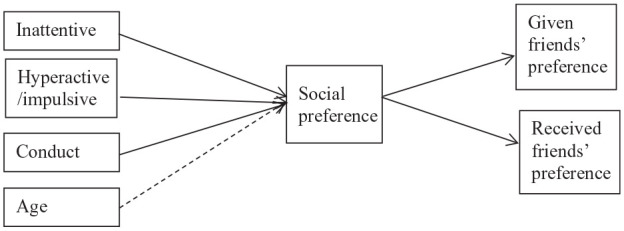
Pathmodel from teacher-rated behaviors via social preference to given and received friends’ preference. *Note.* Intermitted lines are associations that are accounted for but not included in the research questions.

**Figure 2.**
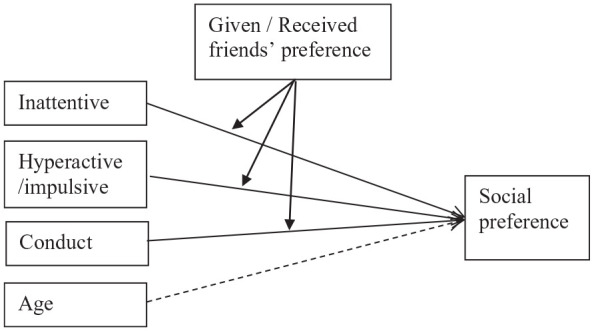
Moderation model of associations between teacher-rated behavioral problems with social preference, moderated by given and received friends’ preference. *Note.* Intermitted lines are associations that are accounted for but not included in the research questions.

To test the limited social resources-hypotheses (hypothesis 2) and the interpersonal contact hypothesis (hypothesis 3), analyses were conducted with the Lavaan package (Rosseel, 2012) of the R statistical program version 4.1.2. (R Core Team, 2021). First, intraclass correlations (ICC) were calculated to assess between classroom and between school variance in the sample. ICCs for preference and friends’ preference were zero, reflecting that the data structure was not nested within classrooms or schools. Therefore, we did not account for nested data. To investigate the hypothesis that higher levels of inattention, hyperactivity/impulsivity, and conduct problems in children were related to having lower preferred friends, via children’s own lower social preference (limited social resources; hypothesis 2), a path model was constructed (see Figure 1). Paths from inattention, hyperactivity/impulsivity, and conduct problems as predictors of children’s social preference were entered in the model. Altogether this yielded a total of six exogenous variables. Furthermore, paths from children’s social preference to both given and received friends’ preference were entered. Following recommendations of Bentler and Chou (1987) we made sure that there were at least five to ten observations per parameter in our model. Model fit was assessed with four model fit statistics: Comparative Fit Index (CFI), Tucker-Lewis Index (TLI), Standardized Root Mean Residual (SRMR), and Root Mean Square Error of Approximation (RMSEA) (Hu & Bentler, 1999). For CFI and TLI a cut-off of .90 indicated adequate fit (Hu & Bentler, 1999). For SRMR and for RMSEA cut-offs of respectively .06 and .08 or less, were utilized (Browne & Cudeck, 1993; Hu & Bentler, 1999). To determine the significance of the indirect effects we used bootstrapping procedures (1,000 bootstrap samples). To investigate whether given and received friends’ preference moderated the behavior – preference associations (interpersonal contact; hypothesis 3; Figure 2), two separate linear regression models (one including interactions with given friends’ status, and one with received friends’ status) were constructed. Each model included the three types of behavioral measures (inattentive, hyperactive/impulsive, conduct problems), both main effects of friends’ preference, three interactions between each of the behavioral measures and friends’ social preference. A power analysis (PWR-package, version 1.3-0, in R) showed that with 9 parameters, 112 participants (power > 0.80, alpha 0.05) we were able to detect medium effect sizes of *f*^2^ = 0.15. These were saturated models and model fit was therefore not assessed. In each of the models age and gender were included as covariates, because behavioral problems as well as peer relations of children with ADHD are known to be related with both age and gender (Ragnarsdottir et al., 2018). However, gender was not significantly related to any of the outcomes, and models with and without gender showed similar patterns of associations. Therefore, we did not include gender in the final models. Missing values were estimated with Maximum Likelihood (missing = ml.x) with robust standard errors.

## Results

### Descriptives

Means and standard deviations of the study variables are displayed in Table 1. Pearson correlations between the study variables are depicted in Table 2. Bivariate correlations between all study variables were below *r* = .6.

**Table 1.** Means (*M*) and Standard Deviations (*SD*) of Teacher-rated Behavioral Problems and Social Preference of the at risk-group, and Comparisons With the Typically Developing Classmates.

Variables	*N*	*M*	*SD*	*t*	*D*
SWAN inattentive	107	1.24	0.80	—	—
SWAN impulsive/hyperactive	107	1.49	0.85	—	—
SDQ Conduct problems	109	2.55	2.04	—	—
Peer ratings: preference	107	−0.61	1.11	−5.73**	0.54
Peer ratings: given friends’ pref.	107	−0.25	1.09	−2.34*	0.23
Peer ratings: received friends’ pref.	94	−0.30	0.83	−3.46**	0.36

*Note.* SWAN and SDQ were only assessed in the at risk-group. SWAN = strengths and weaknesses of ADHD-symptoms and normal behavior; SDQ = Strengths and Difficulties Questionnaire; pref. = preference.

* *p* < .05. **p < .001.

**Table 2.** Correlations Between Demographics, Teacher-rated Behavioral Problems, and Social Preference of the at risk-group.

Variable	1.	2.	3.	4.	5.	6.	7.
1. Age	—						
2. SWAN inattentive	.06	—					
3. SWAN Hyperact./impuls.	−.16	.54**	—				
4. SDQ Conduct problems	.12	.39**	.53**	—			
5. Peer ratings: preference	.12	−.25*	−.19	−.27**	—		
6. Peer ratings: given friends’ pref.	−.11	−.02	−.05	−.16	.22*	—	
7. Peer ratings: received friends’ pref.	−.06	−.04	−.02	−.08	.28**	.58**	—

*Note.* SWAN and SDQ were only assessed in the at risk-group. SWAN = strengths and weaknesses of ADHD-symptoms and normal behavior; SDQ = Strengths and Difficulties Questionnaire; Hyperact./impuls. = hyperactive/impulsive; pref. = preference.

* *p* < .05. **p < .01.

### Hypothesis 1: Differences Between the at-risk group and Their Classmates?

Table 1 displays the results of the comparison between the at-risk group and the group of typically developing classmates on social preference and social preference of their friends. Findings show that children in the at-risk group had significantly lower social preference than their classmates. Furthermore, the classmates that children at risk for ADHD seek out as friends (given friends) as well as peers that chose them as friends (received friends), had significantly lower social preference than average classmates. Additional analyses within the at risk-group revealed that children at risk for ADHD had significantly lower social preference than their given friends (*t*(108) = −2.76, *p* = <.01), but did not differ significantly in social preference from their received friends (*t*(93) = −1.96, *p* = .05). Given friends had on average higher social preference than children at risk for ADHD. Given and received friends did not differ significantly from each other in social preference (*t*(93) = −.45, *p* = .66).

### Hypothesis 2: Limited Social Resources?

Table 3 presents standardized regression weights of the path-model that tested whether higher levels of behavioral problems in children were indirectly related to having lower preferred friends, via children’s own lower social preference (limited social resources-hypothesis). This model fitted the observed data. χ^2^(8) = 4.06, *p* = .85, CFI = 1.00, TLI = 1.15, SRMR = .03, RMSEA = .00 [.00, .06] and explained a total of 24.8% of the variance. Of this, 11.9% of the variance of children’s own social preference was explained by the behavioral predictors. An additional 4.7 % and 8.2% of the variance of given and received friends’ preference respectively, was explained by the child’s social preference. Of the three behavioral measures only conduct problems was significantly related to children’s social preference, with higher levels of conduct problems related to lower social preference. Inattentive behavior was marginally significantly related to social preference (*p* < .10). Furthermore, children’s social preference was related to given and received friends’ social preference. We calculated the bootstrapped indirect effects for the paths from conduct problems to given and received friends’ preference, via own social preference. For given friends’ preference the indirect path did not reach statistical significance, with *p* > .05 and zero just within the bootstrapped confidence interval (*b* = −0.03, *SE* = 0.02, *p* = .144, CI [−0.074, −0.000]. For received friends’ preference the indirect path was marginally significant, and further inspection of the bootstrapped confidence intervals showed that the confidence interval did not include zero (*b* = −0.04, *SE* = 0.02, *p* = .072, CI [−0.081, −0.005]. Given the fact that the bootstrapped confidence interval is a more robust measure this suggests that there is an indirect effect for received friend’s preference. However, explained variance was low.

**Table 3.** Standardized Regression Weights of the Path-model (Hypothesis 2) and the Two Moderation Models for Given Friends and Received Friends (Hypothesis 3).

			Hypothesis 3			
	Hypothesis 2		Moderator: given friends		Moderator: received friends	
Variables	*β*	*p*	*β*	*p*	*β*	*p*
>Predicting own social preference						
SWAN inattentive	−.19	.084	−.24	.**028**	−.24	.**027**
SWAN hyperactive/impulsive	.08	.513	.09	.473	.09	.439
SDQ conduct problems	−.26	.**019**	−.21	.**048**	−.22	.**044**
Age	.17	.074	.22	.**019**	.20	.**025**
>Predicting given friends’ social preference						
Own preference	.22	.**020**				
>Predicting received friends’ social preference						
Own preference	.29	.**003**				
>Main effects of friends’ preference on own preference						
Given friends’ preference			.02	.854	.05	.673
Received friends’ preference			.23	.**042**	.20	.084
>Interactions of behaviors with friends’ preference						
Inattentive × preference			−.20	.082	−.26	.051
Hyperact./impuls. × preference			.14	.287	.09	.513
Conduct × preference			.08	.534	.12	.387

*Note.* Interactions that are reported are interactions with given friends’ preference in the given friends model, and with received friends’ preference, in the received friends model. Significant associations in bold. SWAN = Strengths and Weaknesses of ADHD-symptoms and Normal Behavior; SDQ = Strengths and Difficulties Questionnaire; Hyperact./impuls. = hyperactive/impulsive.

### Hypothesis 3: Protective Effects of Interpersonal Contact?

Table 3 presents standardized regression weights of the two moderation models which tested whether friends’ social preference mitigated the negative effects of children’s own behaviors on their social preference. The models testing moderation of given friends’ preference and received friends’ preference on associations between behavioral problems and children’s own social preference explained 22.4% and 22.7% of the variance in their social preference, respectively. In both moderation models of the four behaviors, conduct problems and inattentive behavior were significantly negatively associated with children’s own social preference, indicating that children with higher levels of conduct problems and inattentive behavior were less preferred by classmates. There were no significant interactions between the behaviors and social preference of given or received friends. However, interactions with inattentive behavior were marginally significant in both models (*p* < .10). Thus, against our hypothesis, associating with higher preferred given or received friends did not buffer against a lower social preference.

## Discussion

Our findings suggested that children at risk for ADHD had lower social preference, as well as given and received friends with lower preference compared to their typically developing classmates. Furthermore, our findings partly supported our “limited social resources” hypotheses, but not our “interpersonal contact” hypotheses. In particular, levels of conduct problems, but not the degree of inattentive and hyperactive/impulsive behavior, was indirectly related to lower social preference of received friends, via their own lower social preference.

### Differences Between the at-risk group and Their Classmates?

Our finding that children at risk for ADHD as well as their unilateral friendships had lower social preference than typically developing classmates, is in line with previous research that suggests that children with clinical levels of ADHD behaviors are more rejected, and that they have fewer friendship opportunities (Cardoos & Hinshaw, 2011; Gardner & Gerdes, 2015; Hoza et al., 2005). In contrast to previous research of Hoza et al. (2005), we found that given and received friends of children with ADHD behaviors also had lower social preference than average in the classroom, while this was not reported by Hoza. These contrasting findings may be explained by differences in study design. While Hoza and colleagues included friends’ preference of one randomly selected comparison child in the same classroom we compared friends’ preference with the average social preference in the classroom, which may be a more robust estimation of the social status of classmates. In sum, even non-clinical levels of ADHD behaviors may already increase the risk for peer rejection, and friendship opportunities for children at risk for ADHD seem to be primarily among other children that are rejected by classmates as well.

Interestingly, we found that given friends had higher social preference than children at risk for ADHD, but received friends did not differ significantly from children at risk for ADHD or from given friends. The first finding suggests that children at risk for ADHD desire to be friends with children who are (slightly) more preferred by classmates (although still less liked than typically developing peers). An explanation may be that children at risk for ADHD might make upward social comparisons with classmates, that is, they might look at opportunities to affiliate with classmates who have higher social status in the classroom (J. K. Dijkstra et al., 2010). Social comparisons can include evaluations of abilities, opinions, self-esteem, and status (P. Dijkstra et al., 2008; J. K. Dijkstra et al., 2010). Potentially, these more highly preferred classmates also show higher levels of prosocial behavior (Rubin et al., 2006). This might make them more desirable as friends. This tendency to seek out friends with slightly higher social preference was also found in a study of Scholte et al. (2009) in victimized youth. The fact that given friends’ preference is still below classroom average suggests that children at risk for ADHD try to become friends with classmates who are not completely out of reach. They might be aware that the most well-liked, socially and academically competent classmates might not be available friendship opportunities.

Indeed, Mikami, Na, et al. (2022) show that classmates who were more socially and academically competent were more likely to dislike children with ADHD. The finding that children at risk for ADHD strive for friends who are not completely out of reach, seems to contrast the large body of literature suggesting that children with ADHD tend to overestimate their own competence and have unrealistically high self-images (positive illusory bias; Owens et al., 2007). As received friends’ did not significantly differ from children at risk for ADHD in social preference, this seems to support the idea that the actual friendship opportunities are among classmates that are closer in social preference. However, this latter difference was still marginally significant, suggesting this finding must be interpreted with some caution. Altogether, our study suggests that children at risk for ADHD struggle in the development of healthy and supportive classroom relationships in similar ways as do children with diagnosed ADHD, and that they make upward social comparisons with classmates regarding social preference.

### Limited Social Resources?

Based on previous research we hypothesized that children at risk for ADHD would have limited social resources. Thus, we expected that higher levels of behavioral problems of children would indirectly be related to having lower preferred given and received friends, via their own lower social preference. Our findings partly supported this hypothesis only for conduct problems. Higher levels of conduct problems were indirectly related to lower received friends’ preference, via own lower social preference, but not to lower given friends’ preference. Although this relationship was not strong, this supports the theory that the friendship opportunities (received friends) of children who are rejected (in relation to their conduct problems) are among other children who are rejected as well. This indirect relationship was not found for inattentive behavior and hyperactive/impulsive behavior, which may be explained by the potential overlap of hyperactive/impulsive behaviors with conduct problems as well as with inattentive behavior. Altogether, these findings may reflect having limited access to social resources to befriend children with a more favorable social position in the classroom (Hartl et al., 2015). Potentially, friends of children at risk for ADHD may be classmates that also show less adaptive behaviors, which could reinforce maladaptive behaviors through altercations between friends (Normand et al., 2022) and be less fulfilling and supportive (see Normand et al., 2023).

Research suggests that mainly popular children in the classroom control social resources (Farmer et al., 2018), as they have much influence on who is liked and who is disliked in the classroom. For classmates, befriending children who are rejected may come with reputational costs, and risks of being rejected themselves (Mikami et al., 2010). This suggests that children at risk for ADHD that are rejected by peers may be stuck in the social position they have in the classroom (Farmer et al., 2018). However, even though children at risk for ADHD might have less friendship options and may particularly relate with other rejected peers, many of them do seem to acquire a friend which is protective in itself (Hoza et al., 2005).

### Protective Effects of Interpersonal Contact?

Regarding our third hypothesis, our findings did not confirm that affiliating with classmates with higher social preference mitigates the negative associations between high behavioral problems and low social preference (J. K. Dijkstra et al., 2010). Even though this was against our hypothesis, this is in line with previous research in typically developing adolescents (Marks et al., 2012) that found that particularly affiliating with a popular peer, more than affiliating with a well-liked peer increased adolescents own social status. Even though in adolescence popularity is more important than for primary school children, it could be that a similar pattern exists in primary school. Because popularity is based on reputation instead on personal evaluation, being associated with a popular peer might also increase the extent to which children are liked by classmates (J. K. Dijkstra et al., 2010). Another explanation might be that in this naturalistic context friends of children at risk for ADHD may not often have above average preference in the classroom and therefore even if friends have slightly higher preference this might not be enough for a protective effect on children’s own social preference. This would be in line with our findings regarding the social preference of friends, who seem to have slightly higher preference, but still below classroom average. Further research into the potential protective effect of being affiliated with popular classmates, as well as on increasing affiliations with more highly preferred peers, might provide valuable insights in how the social context plays a role for the social position of children at risk for ADHD.

### Strengths and Limitations

Our study had several strengths and limitations. First of all, this study is among the first to assess how the social context might influence the social opportunities of children at risk for ADHD. Social preference and friendships are often only seen as a result of children’s social skill deficits and behavioral problems, while the social context also may play an important role in the extent to which children are accepted in classrooms and the opportunities that children have for friendships. Next, we were able to include a sample of children at risk for ADHD in regular education classrooms. This adds to existing research since there are many children that are not diagnosed with ADHD, but might experience similar hurdles in their classroom relationships. Furthermore, we were able to acquire data regarding their social preference and that of their friends, by adding a large control group of classmates that filled out peer nominations.

This study also has some limitations to mention. First, the current study was cross-sectional. Longitudinal data might provide us with a better idea regarding the direction of the effects, as well as regarding changes in social preference over time. Second, the study focus was on behaviors that can be characterized as externalizing, because these tend to be most visibly prominent in social interactions. Future research should consider additional measures, such as comorbid internalizing behaviors. Furthermore, future studies could benefit from adding behavioral measures in the comparison group to increase options for comparison. Along the same line, even though sociometric procedures assessing social preference and friendships are highly reliable and valid measures to assess peer relations (Bukowski et al., 2018), questions about companionship, validation, and self-disclosure could deepen our understanding of the social provisions children have in class (Yust et al., 2023). Adding these behavioral and sociometric measurements might provide us with a more nuanced view on peer relationships of children at risk for ADHD. Third, we assessed the potential protective effect of friends’ preference by utilizing an average preference score of three unilateral friends. These composite scores may move towards the mean, which reduces variance between children and might make it more difficult to capture buffering effects. It might therefore have been better to utilize the preference score of the friend with the highest social preference. We also only assessed ADHD behaviors by teacher reports, while parent and self-reports might provide additive insights beyond classroom behaviors. Finally, the majority of children in our sample were boys (84%), but girls at risk for ADHD are also known to experience much social problems (Kok et al., 2016). Future research would benefit from a more balanced sample.

### Practical Implications

Our findings also have implications for classroom practice. Lately, it is increasingly recognized that the risks of children at risk for ADHD for peer rejection, and their limitations in friendship opportunities, should be seen in light of the social dynamics of the complete classroom and not solely as a result of children’s own deficits (Mikami & Normand, 2015). Previous studies already established that to foster peer acceptance the focus should be on helping children at risk for ADHD steer their behavior in interactions with peers, as well as on improving peer perceptions (Mikami et al., 2017). Our findings support this. Recent classroom interventions that aim to improve the social position of children with ADHD unfortunately show us that targeting improvement of teacher-child relationships and interactions, and modeling inclusive attitudes, is still not enough to foster positive changes in peer acceptance (Mikami, Owens, et al., 2022).

Our study suggests that classmates seem to be reluctant to be associated with children who show ADHD behaviors, partly due to their lower social preference, which might increase the risk for classmates to be rejected by other classmates themselves (reputational costs). Popular children in the classroom (whom are not necessarily highly preferred) are known to be influential in this classroom process, because they set the social norms regarding who is accepted and who is rejected in the group (Farmer et al., 2018). Classroom- and schoolwide interventions that foster prosocial norms and prosocial leadership of popular peers, might enhance social cohesion and reduce exclusion processes. This may provide children at risk for ADHD with more friendship opportunities (Farmer et al., 2018, 2019). Furthermore, even though natural occurring affiliations with more highly preferred peers does not seem to protect against lower preference, it might be worthwhile to actively intervene in increasing familiarity of classmates with children at risk for ADHD. For example, careful rearrangements of seatings in classrooms (e.g., seating the child next to a prosocial classmate) might decrease stigmatizing views and peer rejection (Farmer et al., 2018; Y. H. M. van den Berg & Stolz, 2018). Third, increasing mental health literacy by schoolwide stigma reducing interventions might improve knowledge and attitudes about children at risk for ADHD (Ma et al., 2023).

## Summary and Conclusions

Our findings provided evidence for the hypothesis that children at risk for ADHD have limited social resources. They are less preferred by classmates and their unilateral friends are also less preferred. In fact, higher conduct problems were indirectly related to lower social preference of received friends, via own lower social preference. We did not find evidence for the interpersonal contact hypothesis that being friends with classmates that were more highly preferred could buffer against the negative effects of behavior on low social preference. Altogether, our study adds to the evidence that social opportunities of children at risk for ADHD should be approached and facilitated on a classroom level, instead of only looking at the individual child.

## Supplemental Material

sj-docx-1-jad-10.1177_10870547251349244 – Supplemental material for Social Preference of Children at Risk for ADHD in Schools: Do They Have Limited Social Resources and can Friends Protect Against Peer Rejection? Supplemental material, sj-docx-1-jad-10.1177_10870547251349244 for Social Preference of Children at Risk for ADHD in Schools: Do They Have Limited Social Resources and can Friends Protect Against Peer Rejection? by de Swart F., Veenman B., Oosterlaan J. and Luman M. in Journal of Attention Disorders

## Notes

1. In the Netherlands the WISC-III was used until 2018 when it was followed up by the WISC-V.
