# Supplementary material for: Social Preference of Children at Risk for ADHD in Schools: Do They Have Limited Social Resources and can Friends Protect Against Peer Rejection?
Source: J Atten Disord. 2025 Jun 27;29(10):896–907. doi: 10.1177/10870547251349244 (PMC12255865; doi:10.1177/10870547251349244)
Supplement: sj-docx-1-jad-10.1177_10870547251349244 – Supplemental material for Social Preference of Children at Risk for ADHD in Schools: Do They Have Limited Social Resources and can Friends Protect Against Peer Rejection? [file sj-docx-1-jad-10.1177_10870547251349244.docx]

**Supplement 1.** Sample Characteristics of Participants with Elevated levels of ADHD Problems (N=111)

| Demographic Characteristics |  |
| --- | --- |
| Age (years) | 8.89 (1.85) |
| Gender (% male) | 84% (*n* = 93) |
| IQ | 102.21 (11.10) |
| SES^a^ | 3.32 (0.82) |
| Race (% Caucasian) | 84% (*n* = 93) |
| ADHD diagnosis | 9% (*n* = 10) |
| Other psychiatric diagnosis | 2% (CD: *n* =1; PDD-NOS: *n* =1) |
| Teacher DBDRS  Inattention  Hyperactivity/Impulsivity  ODD  CD | 14.67 (5.53)  15.50 (5.77)  6.24 (4.77)  1.49 (1.76) |
| TTI  Inattention  Hyperactivity/Impulsivity  Combined | 12.50 (5.79)  14.54 (6.05)  27.04 (8.83) |

*Note.* *M* and *SD*s are depicted unless stated otherwise. ADHD = Attention-Deficit Hyperactivity Disorder; CD = Conduct Disorder; DBDRS = Disruptive Behavior Disorder Rating Scale; ODD = Oppositional Defiant Disorder; PDD-NOS = Pervasive Developmental Disorder-Not Otherwise Specified; SES = Socioeconomic Status; TTI = Teacher Telephone Interview. ^a^ SES was measured by parental educational level (average of both parents) through an adapted version of Statistics Netherlands' classification system (1 = primary education, 2 = secondary vocational education, 3 = secondary general education, 4 = undergraduate school, 5 = graduate school; Netherlands Statistics, 2006).
